# “*A PhD is just going to somehow break* you”: A qualitative study exploring the role of peer support for doctoral students

**DOI:** 10.1371/journal.pone.0325726

**Published:** 2025-06-09

**Authors:** Fiona Newlands, Tanvi Markan, Isabelle Pomfret, Emily Davey, Tom King, Anna Roach, Millie Wagstaff, Tom G. Osborn, Roz Shafran, Polly Livermore, Michelle de Haan, Jeanne Wolstencroft, Sophie Bennett

**Affiliations:** 1 Great Ormond Street Institute of Child Health, University College London, Gower, London, United Kingdom; 2 Institute of Psychiatry, Psychology & Neuroscience, King’s College London, London, United Kingdom; 3 Research Department of Clinical, Educational and Health Psychology, University College London, Torrington, London, United Kingdom.; Monash University, AUSTRALIA

## Abstract

Doctoral (PhD) students experience high rates of mental health challenges, including high rates of anxiety, depression, loneliness, and isolation. While universities offer mental health services, these may not fully address the specific needs of doctoral students. Peer support has emerged as a promising adjunct to existing service provision, drawing on shared experiences to provide emotional and practical guidance. This study aimed to explore doctoral students’ perceptions of peer support, identifying their needs and preferences for a peer support programme tailored to the doctoral experience. Nineteen doctoral students were recruited from a university in the south of England and participated in focus groups or semi-structured interviews. Thematic analysis yielded four overarching themes: (1) Barriers to seeking support; (2) Value of peer support for doctoral students; (3) Tailored peer support needs; and (4) Diversity and accessibility. Findings indicate that doctoral students value peer support as a flexible, informal space to share experiences and gain advice from those with similar backgrounds. However, they also emphasised the need for diverse representation among peer supporters, adaptable training to meet neurodiverse needs, and formal recognition of peer supporters’ contributions. Study findings suggest that universities should consider implementing tailored peer support programmes to address the specific challenges faced by doctoral students, incorporating flexibility, cultural sensitivity, and accessibility to create a supportive academic environment. Future research should evaluate the effectiveness of such programs in improving doctoral students’ mental health and well-being.

## Introduction

Research consistently indicates that doctoral (PhD) students experience a higher prevalence of mental health challenges than their peers [1,2]. Evidence suggests they are up to six times more likely to experience anxiety and depression compared to the general population [3], with risks remaining elevated even when compared with age-matched groups [2]. Doctoral students frequently report high levels of loneliness and isolation, often due to the siloed nature of their work and the unique pressures associated with doctoral studies [4]. Additionally, specific groups, such as international students, gender-diverse students and students with disabilities, face even greater challenges, including cultural adjustments, discrimination, and limited support tailored to their needs [5]. In line with these findings, a survey of over 3,000 PhD students in the UK found that over 30% had considered a leave of absence or leaving their studies altogether due to poor mental health [6].

Most universities employ counselling services and while uptake is relatively low [7], many students do not receive adequate support [8]. Barriers such as stigma, fear of academic repercussions, self-reliance and possible long waiting times deter many from seeking help [9,10]. Doctoral students also report that traditional university services are often ill-equipped to address their specific challenges, such as the pressures of publishing, funding uncertainties, and isolation and loneliness frequently experienced by this group [11]. These issues highlight the need for targeted mental health support that specifically addresses the distinct stressors faced by doctoral students.

‘Peer support’ has emerged as a promising adjunct to existing service provision. Defined as the social and emotional support offered by an individual in equal standing [12], this approach provides accessible and often less formal support [13]. Peer support workers are increasingly used to address well-being concerns and tend to assist others by providing either structured behavioural interventions or adaptable, mutual support [7]. Unlike traditional mental health services, peer support programmes leverage the shared experiences of students navigating similar challenges. This is especially important in the context of doctoral students, where isolation and loneliness are key factors contributing to mental health difficulties [6]. The informal nature of peer support often makes it more accessible than formal services, which may be seen as burdensome or stigmatised [14]. Peer support programmes therefore have the potential to fill a critical gap left by university services, particularly in addressing the emotional and practical challenges that doctoral students face.

Existing research on peer support programmes in educational settings, though primarily focused on undergraduate students, suggests that peer support can be highly effective in reducing feelings of distress and promoting well-being [15].

One study adapted the NHS Peer Support Competency Framework [16] for university students and conducted a longitudinal, multi-method evaluation of a peer support programme [15]. The study reported significant reductions in distress and improvements in general well-being among participants. Findings also indicated a need for greater student involvement to improve reach and ensure feasibility and acceptability. While this programme has shown success among undergraduates, its potential to be adapted for doctoral students, who face a specific set of challenges, is an area warranting further exploration.

With this in mind, the aim of the current study was to qualitatively explore doctoral students’ views on peer support and consider how an existing peer support programme could be adapted to meet the specific needs of doctoral students, through a series of focus groups and semi-structured interviews.

## Methods

The study adhered to the Consolidated Criteria for Reporting Qualitative Research (COREQ) guidelines [17].

### Ethics

This study received ethical approval from the UCL Research Ethics Committee (REC Number: 22129/002). All participants provided written informed consent and were fully briefed on the study’s aims, procedures, and their right to withdraw at any time without consequence.

### Participants and Recruitment

Doctoral students were recruited from one university department in the south of England using convenience sampling. Recruitment took place from 1^st^ June 2023–31^st^ August 2023. Recruitment advertisements were distributed through departmental emails, posters, and links to an online survey. Eligible participants were current doctoral students proficient in English. After expressing interest, participants were given detailed information about the study and asked to provide written informed consent. All participants completed a brief screening questionnaire to gather demographic information and previous help-seeking experiences. Participants who were unable or preferred not to join a focus group were invited to participate in an individual interview. Participation was voluntary, and students were reminded of their right to withdraw at any time. All participants provided informed consent and were compensated for their time.

### Data collection

Data were collected through a combination of focus groups and semi-structured interviews. Participants were assigned to focus groups based on their availability. Focus groups and interviews were guided by a semi-structured structured interview schedule developed by a group of doctoral researchers (AR, ED, FN, MW and TK) (see supplementary materials). The topic guide was shaped through iterative team discussions and reflected an exploratory approach. It was also reviewed by members of the project steering group, including researchers and PPIE members including doctoral students and revised accordingly. Questions were designed to assess doctoral students’ previous experiences with peer support, perceptions of an existing peer support training manual, and views of how it could be adapted for doctoral students. Facilitators were doctoral students within the same university department.

Focus groups and interviews were conducted via MS Teams. Focus groups were facilitated in pairs by the doctoral students who were co-investigators on the study. TK co-facilitated two focus groups. FN, ED, AR and MW all co-facilitated one focus group. Interviews were conducted by FN (n = 2), TK, (n = 1), AR (n = 1) and MW (n = 3). Focus groups took place in July 2023 and the semi-structured interviews were conducted between July and August 2023. Focus groups lasted between 1 hour 43 minutes and 1 hour 50 with an average of 1 hour 47 minutes. Interviews ranged from 30 minutes to 1 hour with an average of 41 minutes. All focus groups and interviews were audio-recorded and later transcribed verbatim. Identifying information was anonymised to ensure participant confidentiality.

### Analysis

Participant characteristics are described in the Results section. Data were analysed using reflexive thematic analysis, following Braun and Clarke’s framework [18,19]. This framework was chosen as it supports a rigorous and transparent approach to exploring participants’ perspectives. Initially, transcripts were read multiple times for familiarisation, and preliminary codes were generated through open coding. These codes were then grouped into potential themes, which were iteratively reviewed, refined, and organised into overarching themes. The analysis was conducted using NVivo software to assist with coding and theme development. Two MSc students (TM and IP) independently coded the data, and discrepancies were resolved through discussion and comparison. Themes were further validated by cross-checking with other members of the research team to ensure consistency and credibility. Themes were discussed within the team to ensure that the interpretations accurately reflected the data.

### Reflexivity

Five doctoral 2^nd^-year students (four female and one male) trained in qualitative research conducted the focus groups and interviews. As PhD students within the same institute as participants, the researchers acknowledged the potential impact this could have on participant contributions. Although there were no formal prior working relationships between the student participants or researchers, such as collaborations on other projects, the potential influence of familiarity between some researchers and participants was acknowledged and critically considered throughout the study. To mitigate these effects, the team engaged in continuous reflexive discussions to critically examine how their roles and personal experiences might influence the research process. This included acknowledging any preconceptions they might hold and how these could influence their questions and responses during the interviews. Meetings were held with the project leads and the study steering group to discuss preliminary codes and themes during the focus groups and interviews. A semi-structured topic guide was used to support consistency and minimise interviewer bias and focus groups were facilitated by multiple researchers to promote balanced discussion and reduce the influence of any one individual. Additionally, formal analysis was conducted by the MSc students (TM and IP) with no prior connection to participants to mitigate the impact of existing relationships between student researchers and participants. Regular meetings between TM, IP, and two of the doctoral student researchers (FN and ED) provided a space to discuss emerging themes, raise questions, and reflect on the analytic process. This approach was intended to support reflexivity and ensure a diversity of perspectives, while maintaining the independence of the MSc students’ analysis. This reflects the principles of reflexive thematic analysis, which, rather than aiming to reach consensus, values collaboration as a way to support richer and more thoughtful interpretation of the data [20].

## Results

### Participant characteristics

The study included 19 doctoral students. Participants’ ages ranged from 24 to 66 years (Mean: 32.5, SD = 10.8), with a majority identifying as female (68.4%). Participants were majority White British (n = 11), but other participants self-identified as from other White background (n = 3), Chinese (n = 3), any other Asian background (n = 3), African (n = 1), or did not state their ethnicity (n = 1). A majority of participants were domestic (UK) students (n = 11) compared with those who were international students (n = 8). The sample’s demographic details are presented in Table 1.

**Table 1:** Summary of demographic characteristics.

Demographics	Description	Number	Percentage
**Age**	20-30	13	68.4%
	31-40	3	15.8%
	41-50	1	5.3%
	50-above	2	10.5%
**Gender**	Male	6	31.6%
	Female	13	68.4%
**Ethnicity**	White British	10	52.6%
	Any other White background	3	15.8%
	Chinese	3	15.8%
	Any other Asian background	1	5.3%
	African	1	5.3%
	Ethnicity not stated	1	5.3%
**Year of Study**	Year 1	5	26.3%
	Year 2	7	36.8%
	Year 3	6	31.6%
	Year 4	1	5.3%
**Fee Status**	Funded by the Institution	11	57.9%
	Self-funded	8	42.1%
**Residency Status**	Domestic	11	57.9%
	International	8	42.1%

**Fig 1:**
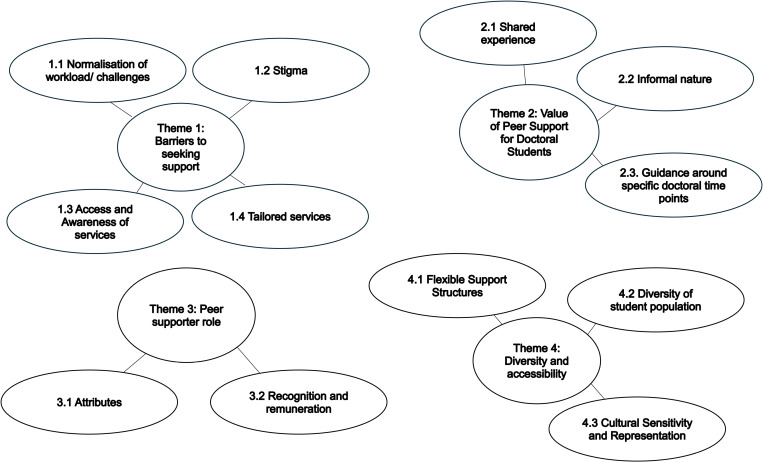
Thematic Map.

Four overarching themes and thirteen subthemes were identified through thematic analysis: (1) Barriers to seeking support; (2) Value of peer support for doctoral students; (3) Peer supporter role; and (4) Diversity and accessibility. See Fig 1 for a thematic map.

### Theme 1: Barriers to seeking support

Participants highlighted several challenges experienced through their doctoral studies that impacted their mental health and well-being including issues around loneliness and concerns about finances. Despite the challenges, several barriers were identified that prevented students from engaging in the support available.

#### 1.1. Normalisation of workload/ challenges.

Participants described how they struggled with the difficulty and volume of doctoral work and competing deadlines, “*where you’ve got so many things to juggle*” (PP9). Managing these competing deadlines not only contributed towards a worsening of their mental health but also meant students felt they did not have the time to access support and de-prioritised their well-being:

*“Also, time constraints. If you feel like it’s just not feasible because of other priorities that you have… ultimately, you are, I guess, forfeiting [or] de-prioritising your mental health in the sense of those other aspects.” (PP3)*

Struggles were considered an inherent and expected part of the doctoral experience, with many believing that “*a PhD is just going to somehow break you*” (PP17). Participants shared that this normalisation of suffering discouraged them from seeking formal support:

“*we normalise the struggles of a PhD student a lot. Everyone is meant to suffer. It’s meant to be hard [...]. How many times I’ve heard, “Oh, I didn’t get through my PhD without having a mental breakdown.” So, I think everyone assumes that everyone is struggling, so no one seeks help*.” (PP17)

Participants reflected on the prevalence of self-doubt and imposter syndrome amongst doctoral students. One participant explained how the pressure to produce outputs including journal publications led to feelings of inadequacy:

*“If you haven’t made Forbes by your 30s, you are not worth, right? […] students are being raised in a culture that really pushes you to show that you perform, show that you’re achieving things.”* (PP5)

There was a sense that engaging with peers could be a helpful way to validate their experiences and reduce feelings of inadequacy. As one participant noted, peer interactions reaffirm that “*the way you’re feeling, everybody will be feeling, at some point in the PhD*” (PP16).

#### 1.2. Stigma.

Participants reported they felt stigma surrounding seeking mental health support. Participants described hesitating to seek support out of fears that the situation would escalate to an unwanted degree and expressed concern about issues “*becoming a bigger deal than you want it to be”* (PP6). Participants also worried about the potential damage to their academic reputation, reflecting, *“you don’t want it [your problems] to affect other people’s views of you or your reputation.”* (PP15)*.*

There were also concerns around confidentiality and whether trying to seek support would have repercussions:

*“You don’t want it to be flagged up and raised, and it’s suddenly a really big issue, and everyone has to be called in, and you get labelled as a problem, I think that would prevent me, the fear of stigmatising it, I guess.”* (PP6)

#### 1.3. Access and awareness of services.

Doctoral students were aware university support services existed but struggled with locating and accessing them and felt information about these services was either not readily available or was poorly communicated by the university:

*“I think the hardest thing is, where do you find it and how do you start on?”* (PP11)

With regards to peer support, participants shared that a lack of clear communication about the purpose and benefits of peer support deterred their engagement:

*“If the person that’s supposed to be accessing it doesn’t fully understand what it is they’re accessing, and why, then they may not access it, for those reasons, because they don’t really understand what the purpose of it is.”* (PP15)

Those who could locate services experienced challenges in accessing them. Participants felt that services were not accessible or flexible enough to accommodate for their needs or preferences. One participant described that they “*needed face-to-face counselling*” but the university services had *“limited availability because of the COVID issues”* (PP7). High demand meant appointments were rare and difficult to access particularly for doctoral students with busy schedules:

*“But obviously, the issue is currently it needs to become more available. Because it’s also quite hard to get access to it because a lot of people are trying to get access to it.”* (PP3)

#### 1.4. Tailored services.

Finally, there was a sense amongst doctoral students that the services on offer, including peer support, were not tailored to their specific needs and often felt “*very impersonal*” (PP3). One participant felt this generic nature of support deterred their engagement:

*“it is not tailored to what you need [...] There is peer support but that exists specifically overall, that does not specifically exist within PhD-to- PhD level, that I think, for that reason, you may find you’re less inclined to reach out*.” (PP3)

The absence of peers with similar backgrounds meant students were less likely to engage any such programme as they felt their experiences would not be understood by others:

“*I’ve been working eight years in a professional capacity. So, I don’t always feel as though I’m at the same [level as others]. There aren’t that many peers around me who have a similar background*.” (PP6)

### Theme 2: Value of peer support for doctoral students

Despite barriers to accessing formal support, participants highlighted numerous benefits and opportunities provided by peer support. Through shared experiences and specific guidance during critical doctoral milestones, peer support was highlighted as a valuable resource.

#### 2.1. Shared experience.

Participants expressed that they would benefit from speaking to peers who *“have similar experiences or similar struggles or feel similar pressures”* (PP17)*.* By speaking about and relating to these shared experiences, peers would be able to normalise the struggles of those being supported and offer helpful advice:

*“I imagine a peer support worker is someone who is more in your situation. So, if there’s something that maybe they’ve been through themselves, they might be open to providing some advice or talking about what they’ve been through, maybe to help you.”* (PP12)

*“So, I guess students would benefit from knowing that other students have similar experiences or similar struggles or feel similar pressures.”* (PP17)

There was a sense of comfort knowing other people have had similar experiences, as “*it’s a lot less stressful knowing people have already done it*” (PP16). One participant felt that peers could offer more relatable insights than supervisors:

“*I think [peer support] would give a PhD student a chance to talk about issues that they don’t necessarily want to raise with their supervisor. And I think that would be really beneficial for someone to be able to talk to someone who would understand what they’re going through*.” (PPI2)

Another participant illustrated the significance of shared experiences with a personal instance from their doctoral studies:

“*I know my friends who don’t do PhDs think I’m crazy because I do cells. Then, I’ll come back home and be like crying because all my cells have got contaminated and [I would] not have people that understand what it means. Because it’s two months of work for me that I’ve lost, but only someone who does what I do, would understand what it would mean*.” (PP16)

Some participants advocated for a ‘buddy system’ approach to peer support, whereby peers are paired based on similar experiences or academic pursuits. They believed this approach would create a shared connection, as “*really what you’re looking for is a buddy*” (PP10).

#### 2.2. Informal nature.

Participants reflected that they valued the informality and relaxed nature of peer support. One participant felt that formal support was *“rigid”* and limited to *“certain communication styles or certain frameworks”,* making it *“harder to share”* (PP12)*.* Peer support was an alternative forum for handling *“general or minor stress or issues or challenges”* while avoiding the stress and intensity of a *“big formal process”* (PP12)*:*

*“I think not having it in such a strict way, or manner. When we talk about peer support, it’s just literally- it may be a bit like the environment itself doesn’t have to be so formal. Because basically, ultimately, you’re just having a conversation.”* (PP3)

Peer support offered a space where students could express themselves freely, making it easier to discuss challenges in a way that felt natural and supportive.

#### 2.3. Guidance around specific doctoral time points.

Participants noted that there were specific times during their studies during which they were more likely to experience significant stress and uncertainty, such as upgrades and viva examinations. One participant highlighted the heightened stress levels around some major milestones:

*“When you’re prepping for things like your upgrade, that’s also a point where you have extra stress and obviously it makes more sense to access it then.”* (PP11)

*“I’m coming up to my viva, so I probably will seek out some peer support and advice on the viva, another big aspect of the PhD.”* (PP1)

Participants shared that support from peers during these critical periods was highly

valued. One student commented on the importance of peer support at the start of their studies and how it would be beneficial to answer questions and alleviate concerns during this time, as well as a convenient way to socialise and mingle immediately:

*“Especially in the beginning, because I think it’s the most uncertain point of your PhD, like just at the start, when you kind of know what you’re doing but you actually don’t know. So, it’s just nice to have this support, to know that you’re not alone in this feeling.”* (PP7)

### Theme 3: Peer supporter role

Throughout the interviews and focus groups, participants identified key characteristics and qualities they would value in a peer supporter, such as practical problem-solving abilities, empathy, and active listening skills. Additionally, participants discussed the need for formal recognition and compensation for peer supporters, acknowledging the demanding nature of the role and the time commitment it entails.

#### 3.1. Attributes.

Participants expressed that they would like a peer supporter who was able to give advice and problem-solve effectively with day-to-day issues, not just mental health and well-being:

“*Essentially, peer support can help you solve your problems. Like problems that are in your everyday routine work, or little struggles or frustrations you have, you can- It is a way that you can start understanding, ‘Oh, I can actually solve things by discussing things and finding solutions far faster’.”* (PP5)

It was felt that advice and signposting would be particularly helpful for international students, who may lack information about navigating healthcare systems:

*“I need some vaccination…but I have no idea how I can get that vaccine in the UK. I have no experience about this, so if there can be local students, students who are familiar with the GP things, the NHS things or the vaccine things it can be much more easier for me.”* (PP2)

Participants discussed how they would benefit from speaking to an empathetic and supportive peer supporter:

*“Yeah, I guess communication skills would be really important. Empathy and active listening, summarising, reflecting. A nonjudgmental approach, which I guess is linked with empathy and trustworthiness.”* (PP1)

#### 3.2. Recognition and remuneration.

Participants stressed the need to formally acknowledge the time and effort invested in peer support training and activities. They noted that doctoral students, who are already managing substantial academic and personal responsibilities, often struggle to allocate additional time for training. Sharing their personal experience of being a peer supporter, one participant highlighted the demanding nature of this role: *“We were just given way too much work, way too many students, way too much responsibility, emotional and practical*.” (PP17).

Participants emphasised the need to recognise their time and investment to motivate participation:

“*Most people end up doing that totally for free and yes, other people are benefiting from it, and you’re benefiting from it, and it’s a learning environment, but it doesn’t help you get forward with your [PhD] project*.” (PP4)

Participants shared that without formal recognition or a supportive culture, doctoral students are less likely to engage in peer support or become a peer supporter:

“*there’s no real validation for them to be a well-being champion. Why would a PhD student just off their own back do that when they’ve got all the other stresses that they have?*” (PP4)

“*If it can be accredited in some ways, I always like those courses, because even though it sounds a bit negative to be like, ‘I’m not doing it unless there’s a point to it’, but some sort of accreditation that you can say, ‘This is something I’ve done’, and that you can put it on your CV in the future and use it as, ‘Actually, this counted as doing one module of this course’, or whatever, always good as well.”* (PP6)

Some participants proposed offering monetary compensation to incentivise and encourage participant in peer support programme from the perspectives of a peer supporter. They felt that doctoral students are generally underpaid, making it unfair to expect them to provide peer support without adequate compensation. One participant emphasised that, while doctoral students are willing to help, financial support would help them manage their commitments while supporting peers:

“*I do think that students in general are not paid enough and then to ask them to provide that support is [wrong]. Every PhD student I’ve ever met has been wonderful, and they would do it gladly if they had enough resources. But we have our PhDs to do as well, and I do think that money helps*.” (PP17)

However, some participants cautioned that offering financial compensation might overshadow the altruistic motive behind participation:

“*I think it should be a small sum because you don’t want someone joining this role just because of the money. So, you don’t want someone getting in a room, sitting there, listening to the other, offering nothing and just getting paid*.” (PP5)

Participants suggested that financial compensation should be an interim measure until broader systemic improvements are made, such as better support from faculty and integration with existing well-being services:

“*until we’ve changed the environment to be better for PhD students, their supervisors are supporting them better, and they’re accessing services because student well-being services have become a more accessible place, until then I do believe that we should pay people for their emotional and practical time*.” (PP17)

### Theme 4: Diversity and accessibility

In discussing peer support, participants highlighted the critical need for diverse and accessible support structures that cater to the varied backgrounds, needs, and schedules of doctoral students. Many noted that a one-size-fits-all approach does not account for the unique challenges faced by students with different personal, cultural, and academic circumstances. Flexibility, cultural sensitivity, and inclusivity emerged as essential elements to ensure that peer support is accessible and meaningful for all students. Participants also emphasised the importance of flexible support formats, adaptive training for neurodiverse students, and cultural representation in peer support roles.

#### 4.1. Flexible Support Structures.

Participants highlighted a strong preference for flexible modalities to accommodate the diverse schedules and needs of doctoral students. Many participants advocated for hybrid delivery formats, allowing a choice between in-person and online sessions.

This flexibility would accommodate the varied personal preferences and comfort levels of doctoral students:

“*Equally maybe offering them [peer support services] hybrid for people who are more worried about being there in person and stuff like that or having in person as well as just online sessions, so people depending on their level of comfort, they attend one versus the other*.” (PP11).

Participants noted that a hybrid approach is particularly beneficial for students who are remote, part-time, or have conflicting schedules. As one participant observed, accessing face-to face peer support or training can be more challenging for these students:

“*I guess it’s also important too, more in the area where people are working from home so much. [...] I do see that, if you’re working from home, I guess, it’s harder to see people all the time as well.”* (PP16)

#### 4.2. Diversity of student population.

Participants reflected on the need for accessible and flexible peer support to accommodate for the diverse support needs of doctoral students. Two participants spoke about how they felt their university was currently failing students with additional needs and disabilities so this would need to be incorporated in peer support.

*“Probably, also, there is a lot of evidence that [University] does not prioritise people with additional needs…So, partly from my involvement in the equality and diversity inclusion group, there are many gaps in which we are failing people. Like, some lecture rooms still doesn’t have ramps, for example, after this many years.” (PP8)*

Therefore, peer support groups should be held in an accessible space when in person, or be held online and *“have subtitles”,* which would benefit *“international people or people with hearing disabilities”* (PP11)*.*

Participants also emphasised the need for training content to be adaptable for

neurodiverse students. For instance, one participant illustrated how conventional training on topics such as active listening might not be suitable for neurodiverse students:

“*I used to work with children with autism, ADHD and [...] I think that telling people what an active listener is, is very neurotypical, and I think that making eye contact is really hard for some people. I think that we shouldn’t ostracise or limit the availability for people with autism to be peers.”* (PP17)

#### 4.3. Cultural Sensitivity and Representation.

Participants stressed the importance of addressing cultural diversity in peer support sessions and training materials. They noted that cultural differences greatly impact how students seek and respond to support. Consequently, participants emphasised that effective peer support requires an understanding of and sensitivity towards these differences:

“*If you’ve got people who come from totally different backgrounds, but what if the person who you’re trying to support doesn’t need support in the way that you need support? So, you don’t really know what to offer and what to suggest.”* (PP4)

To address these cultural differences, participants recommended recruiting peer

supporters from diverse backgrounds. They noted that diverse representation could potentially enhance students’ relatability and engagement with the peer support program. Participants suggested pairing students with peers from similar backgrounds to ensure more effective support:

“*I think we’re coming from a very Eurocentric, white, neurotypical perspective. I think you should have it that neurodiverse people are able to be a peer for other neurodiverse people, and black or Asian students can be peers specifically to black and Asian students, if that is what they want*.” (PP17)

These findings highlight the critical need for diversity and accessibility in peer support structures for doctoral students. By offering flexible formats, accommodating diverse support needs, and fostering cultural sensitivity, universities can better address the varied experiences and challenges faced by their doctoral students.

## Discussion

This study explored doctoral students’ perspectives on peer support and how it could be adapted to meet their specific needs. Findings from this study align with existing research highlighting the mental health challenges faced by doctoral students, including stress, isolation, and imposter syndrome [1,2]. While universities offer a range of mental health services, access can be uneven, with students often navigating complex systems and relying on informal support networks due to stigma, cultural barriers, or service limitations [21]. Peer support, as described by participants, offers a flexible, inclusive, and relatable option that may help reduce barriers to accessing support.

One key theme identified from the focus groups and interviews was the importance of shared experiences in peer relationships. Participants emphasised that having a peer who had encountered similar challenges could normalise their struggles and provide relatable advice. Many participants expressed the desire for peer support for day-to-day challenges rather than exclusively issues relating to mental health and well-being. Such support suggests peer support could prevent the development or deterioration of mental health problems. The emphasis on shared experience echoes research on peer support in mental health and behavioural health contexts, which suggests that relatability, whether through shared role, identity, or experience, is central to building trust and reducing stigma [22]. This is particularly important in doctoral settings, where students may hesitate to disclose difficulties in hierarchical academic relationships [23]. Peer support offers an informal environment that can reduce the stigma associated with seeking help, making it particularly appealing for doctoral students who may hesitate to engage with formal university services due to fear of judgement or academic repercussions [9,24]. This aligns with the broader literature on peer support, which suggests that informal, peer-led initiatives can effectively bridge gaps left by traditional services by offering an approachable and non-hierarchical form of support [13].

Moreover, participants underscored the need for diversity and inclusivity within peer support programmes. The study revealed that while a shared doctoral experience is valuable, peer support must also account for additional layers of diversity, including cultural background, academic discipline, and specific challenges faced by international and neurodiverse students. Previous studies have highlighted that these factors can intensify feelings of isolation and create barriers to accessing support, which further highlights the importance of culturally sensitive and accessible peer support. For example, Hu and Flynn report that international students, LGBTQ+ students, and those from minority ethnic backgrounds often face additional cultural and systemic obstacles when seeking help [25]. Similarly, Syharat et al. found that neurodivergent graduate students frequently feel pressure to conform to neurotypical expectations and may self-silence in academic relationships to avoid jeopardising their progress, which can contribute to increased isolation and unaddressed mental health needs [26]. Such findings highlight the importance of developing peer support approaches that are grounded in shared experience and culturally sensitive, inclusive, and adaptable to diverse student needs. Reflecting this, participants in the current study recommended hybrid models of peer support offering both in-person and online options to enhance accessibility and provide flexibility for students navigating different schedules, responsibilities, and preferences.

Another critical consideration was the structure and training of peer support programmes. While peer support is inherently informal, participants noted the value of training that encompasses key skills such as active listening, empathy, and confidentiality. However, they also stressed that training should be adaptable to accommodate neurodiverse individuals who may require alternative approaches to communication and engagement. Research has similarly highlighted the importance of adapting peer support for diverse student populations and ensure that support mechanisms do not inadvertently exclude certain groups [27]. Training content should be inclusive and adaptable, promoting accessibility without compromising the effectiveness of the support provided.

Finally, participants highlighted the need for formal recognition and compensation for peer supporters. While previous research has noted the intrinsic rewards of peer support, such as skill development and a sense of community [28], participants emphasised that formal recognition, such as accreditation or monetary compensation, could significantly enhance participation. This finding reflects a practical consideration, particularly given that many doctoral students are under financial strain and may struggle to justify the time commitment required for peer support roles without adequate compensation.

### Implications for practice

The findings of this study suggest several recommendations for implementing effective peer support programmes for doctoral students within the context of the university studied. Participants highlighted the value of support structures tailored to the doctoral experience and adaptable to diverse student backgrounds and needs. A hybrid delivery format, offering both in-person and online options, may enhance accessibility by allowing students to choose the mode of participation that suits their comfort and availability. Additionally, formal recognition of peer supporters’ efforts—whether through accreditation, compensation, or professional development—may help to build a sustainable and engaged peer support network. The findings are drawn from a single institutional context and were initially intended to inform practice at the university studied. Nonetheless, they may offer a useful basis for further exploration or adaptation incomparable contexts.

### Limitations and future research

A limitation of this study is its focus on a single department and one university, which may limit the generalisability of findings. The use of convenience sampling to recruit participants may have introduced selection bias, as those with particularly negative experiences or struggles may have been more motivated to participate. Findings may over-represent the perspectives of students experiencing greater challenges. Additionally, as the researchers and participants were all PhD students within the same wider department, it is possible that this shared context influenced how comfortable participants felt sharing their views, which may have shaped their responses. Additionally, the broad age range of participants (24–66 years) may not reflect the typical demographic profile of PhD students. Future research could explore the effectiveness of peer support models across various institutions and examine how specific programme adaptations impact mental health outcomes for doctoral students. Future studies could also evaluate the effectiveness of peer support programmes by assessing their impact on both academic progression and well-being among doctoral students.

## Conclusion

This study contributes to the growing body of literature on peer support for doctoral students by identifying key elements that make peer support feasible, effective, accessible, and meaningful for this population. As universities increasingly recognise the mental health challenges of doctoral students, tailored peer support programmes can play a crucial role in addressing these needs, creating a supportive academic environment that promotes both personal and professional development.

## Supporting information

S1 File Supplementary materials. (DOCX)
