# Supplementary material for: “A PhD is just going to somehow break you”: A qualitative study exploring the role of peer support for doctoral students
Source: PLoS One. 2025 Jun 9;20(6):e0325726. doi: 10.1371/journal.pone.0325726 (PMC12148168; doi:10.1371/journal.pone.0325726)
Supplement: S1 File — (DOCX) [file pone.0325726.s001.docx]

**Supplementary materials- Topic guide**

| **Question** | **Prompt** |
| --- | --- |
| **Expectations from a peer support programme** | |
| What do you understand by peer support? | What type of support would you expect peer supporters to offer?  Who would you consider a peer to be? |
| When, if at all, would you access a peer support programme? | E.g., after a particular point in time during your PhD |
| How, if at all, do you think a PhD student benefit from this form of support? |  |
| What, if anything, do you think would be a challenge/ disadvantage of accessing this type of support? |  |
| **Peer support training** | |
| How would you expect a peer supporter to be trained? | What topics might you expect to cover?  What format would you expect it to be in?  How long would you expect it to take? |

| ***Views on seeking help - Tom*** | |
| --- | --- |
| If you felt you needed help for your mental health and wellbeing, where would you go in university to access this and why? |  |
| What type of support would you want them to offer you? | Psychological, practical support etc. |
| What would prevent you from seeking support for your mental health at university |  |

| **Overview of existing training** | |
| --- | --- |
| Have you heard of training before? | If yes, can you tell me a bit about what you know about it |
| What are your initial thoughts on the training package? | Anything that surprised you?  Anything else you would expect to be covered in the sessions?  Anything you think would be particularly helpful/ unhelpful?  How could the training be improved? If so, how could it be improved? |
| What, if anything, would make the training more relevant to PhD students? |  |

| **Views on existing training** | |
| --- | --- |
| What are your thoughts on training materials? | Anything you like?  Anything you dislike?  Anything that could be improved? If so, how could it be improved?  Anything that would make them more accessible? |
| **Training from the perspective of mentees**  i.e., the person receiving the peer support | |
| What, if anything, would help or encourage you to access peer support? |  |
| What barriers, if any, are there to you participating in peer support? |  |
| **Training from the perspective of mentors**  i.e., the person delivering the peer support | |
| What, if anything, would encourage you to undertake training as a peer supporter for other PhD students? |  |
| What barriers, if any, are there to you undertaking the peer support training? |  |
